# A comparison of soft palate thickness, length, and volume in bulldogs before and after staphylectomy or folded-flap palatoplasty: A prospective clinical study

**DOI:** 10.1371/journal.pone.0362626

**Published:** 2026-11-13

**Authors:** Eva A. Ruzics, Cristina Piedrahita Velasquez, Penny J. Regier, Diego A. Portela, Kathleen M. Ham, James Colee, Federico R. Vilaplana Grosso

**Affiliations:** 1 Department of Small Animal Clinical Sciences, University of Florida, Gainesville, Florida, United States of America; 2 Oklahoma Veterinary Specialists, Tulsa, Oklahoma, United States of America; 3 Department of Comparative, Diagnostic, and Population Medicine, University of Florida, Gainesville, Florida, United States of America; 4 Institute of Farm and Agricultural Sciences, Statistics Consulting Unit, University of Florida, Gainesville, Florida, United States of America; Dakota State University, UNITED STATES OF AMERICA

## Abstract

**Objectives:**

To compare soft palate (SP) length, thickness, and volume in French bulldogs and English bulldogs before and after staphylectomy or folded flap palatoplasty (FFP), and to compare the success of procedures in reducing these dimensions.

**Materials and Methods:**

Dogs diagnosed with brachycephalic obstructive airway syndrome (BOAS) underwent staphylectomy (n = 8) or FFP (n = 8). Pre-operative and 4-week post-operative computed tomography was performed to assess SP dimensions and evaluate surgical outcomes.

**Results:**

Both procedures resulted in a reduction of SP length (staphylectomy mean reduction = 0.91 cm, 95% CI [0.44 cm, 1.39 cm]; FFP mean reduction = 0.86 cm, 95% CI [0.39 cm, 1.33 cm]), and volume (staphylectomy mean reduction = 2.81 cm^3^, 95% CI [1.36 cm^3^, 4.27 cm^3^]; FFP mean reduction = 2.54 cm^3^, 95% CI [0.84 cm^3^, 4.24 cm^3^]) when comparing pre- and post-operative dimensions within each procedure group. Staphylectomy did not result in SP thinning, while FFP resulted in a mean thinning of 0.30 cm (95% CI [0.10 cm, 0.50 cm]). There was no difference in the degree of SP shortening (adjusted mean difference 0.01 cm, 95% CI [−0.33 cm, 0.35] cm) or reduction of SP volume (adjusted mean difference 0.32 cm^3^, 95% CI [−0.52 cm^3^, 1.16 cm^3^]) between procedure groups. Folded flap palatoplasty resulted in greater SP thinning than staphylectomy.

**Clinical Significance:**

Both staphylectomy and FFP resulted in a reduction of length and volume of the SP. FFP resulted in SP thinning, while staphylectomy did not. In dogs with concurrent SP thickening and elongation, FFP may be the preferred surgical treatment.

## Introduction

Brachycephalic obstructive airway syndrome (BOAS) is a condition affecting brachycephalic dogs that is characterized by several anatomic anomalies of the upper airway [1–3]. Primary upper airway anomalies include stenotic nares, aberrant nasal turbinates, relative macroglossia, elongated soft palate (SP), and tracheal hypoplasia [1–5]. An elongated SP is defined as extending caudal to the tip of the epiglottis and/or palatine tonsils [1]. This is the most frequently noted anatomic feature of BOAS, with 87.1%−97.6% of dogs with BOAS affected [1,2,6]. Secondary changes, including hyperplastic tonsils, SP thickening, everted laryngeal saccules, and laryngeal collapse, develop due to the excess negative inspiratory pressure generated by primary features [1–4,7]. Thickening of the SP has been described in dogs affected by BOAS [8–10]. The presence of a hyperplastic SP results in obstruction of the rima glottidis and additional nasopharyngeal obstruction [3,9,11]. In sum, these abnormal features result in increased upper airway resistance, manifesting in stertorous breathing, exercise intolerance, dyspnea, and gastrointestinal (GI) signs [1–3,12].

Traditional surgical management of this condition includes rhinoplasty, staphylectomy, and laryngeal sacculectomy [1,3]. It is worthwhile to note that traditional surgical management of BOAS may in some cases result in sub-optimal outcomes due to the presence of prominent alar folds, aberrant obstructive nasal turbinates, and thickened soft palates that are not addressed in traditional techniques [13–16]. Modified multilevel surgical techniques, including ala-vestibuloplasty, laser-assisted turbinectomy, and folded flap palatoplasty (FFP) may be particularly relevant in dogs with extreme brachycephaly affected by these conformational abnormalities, and have been shown to result in improved outcomes as compared to traditional multilevel surgery [16].

Staphylectomy and FFP are two procedures used to surgically address the SP in dogs affected by BOAS. Staphylectomy is the more conventional technique used to shorten the SP, in which the caudal portion of the SP is resected. Landmarks for the extent of palatal resection are at the level of the cranial to mid palatine tonsils. Folded flap palatoplasty was first described by Findji and Dupré in 2008 as a technique intended to simultaneously shorten and thin the SP [17]. By addressing both SP length and thickness simultaneously, FFP may offer a theoretical advantage over staphylectomy by improving obstruction at both the rima glottidis and nasopharynx. When performed correctly, it is expected that both procedures would result in a similar degree of soft palate shortening.

The primary objective of this study was to compare pre- and post-operative dimensions (i.e., length, width, and volume) of the SP in brachycephalic dogs undergoing staphylectomy or FFP for management of BOAS. We hypothesized that FFP would result in greater SP thinning compared to staphylectomy.

## Materials and methods

### Study design

This was a single-center, parallel-group study conducted in the United States.

### Study subjects and inclusion criteria

The present study was approved by the University of Florida Institutional Animal Care and Use Committee (IACUC #201810347) and by the University of Florida Veterinary Hospital Research Review Committee.

After obtaining informed owner consent, French and English bulldogs presented for evaluation of BOAS and undergoing surgical upper airway correction were prospectively enrolled between December 2019 and September 2022. Dogs between 6 months and 5 years old and no history or evidence of other systemic diseases were included. A thorough history and physical examination were obtained for each dog. Dogs were excluded if they had a history of prior upper airway surgery, facial trauma, grade 3 laryngeal collapse, normal SP length, or evidence of lung pathology, including aspiration pneumonia. Each treatment group comprised 8 dogs and the groups were not randomly assigned. Dogs were assigned to undergo either FFP or staphylectomy based on the attending surgeon’s procedural preference, prior to airway examination. Computed tomographic (CT) imaging and airway examination were performed immediately prior to surgery.

### Procedures

Dogs had intravenous catheters placed in either their cephalic or saphenous vein to maintain venous access. Patients received dexmedetomidine (5–10 µg/kg) and underwent a pre-operative CT examination of the head, neck, and thorax under the same sedation event.

Computed tomography examination was performed under conscious sedation without intubation. Dogs were supplemented with oxygen via a face mask for 5 minutes prior to sedation. Intravenous Lactated Ringer’s Solution was provided at 5–10 ml/kg/hr during the scan. Lead II electrocardiography, blood pressure, and pulse oximetry were monitored throughout the sedation. Glycopyrrolate (0.01 mg/kg) was administered as needed on a case-by-case basis for clinically significant bradycardia as determined by a board-certified veterinary anesthesiologist (DAP). Dogs were positioned in sternal recumbency with the neck extended and forelimbs slightly abducted. The mouth was held open with a spacer made up of a 3 mL syringe casing positioned between the maxillary and mandibular canine teeth, allowing visualization of individual airway structures, as described by Liu et al. [18], and the tongue was gently pulled rostrally to its maximum extent, then left to relax.

All CT scans were performed using a 160-slice (Aquilion Prime) multidetector row, helical CT unit (Toshiba Medical Systems, Inc., Tustin, California). Scans were performed in helical acquisition mode with a scan thickness of 0.5 mm, a pitch of 0.8, 100 mA, 120 kVp, tube rotation time of 0.5 s, and a 512 × 512 matrix. All dogs received 2 mL/kg iohexol (300 mgI/mL – total dose 600 mg/kg) (Omnipaque; GE Healthcare Inc., Cork, Ireland) injected intravenously using a power injector (Stellant, Medrad Inc., Warrendale, Pennsylvania). The injection flow rate was set at 2 mL/s, with a pressure limit of 325 psi. Postcontrast images were acquired approximately 30–40 seconds after intravenous contrast administration, with some variability in the timing throughout the study period. Images were reformatted into transverse, sagittal, and dorsal planes using soft tissue and bone algorithms. The slice thickness of the reformatted images was variable, between 1 and 3 mm, and the display field of view was tailored to patient size. Each study was reviewed using a commercially available imaging software, Horos (The Horos project, Purview, version 3.3.6). Images were stored on a picture archive and communication system.

Following the initial CT scan, anesthesia was induced with either alfaxalone (0.9 mg/kg) or propofol (0.9–7.5 mg/kg) administered IV to effect. Dogs were intubated and maintained under general anesthesia with either sevoflurane or isoflurane. Anatomic airway examination was performed similarly by all clinicians using a standard airway examination form and performed following CT examination and prior to endotracheal intubation. Airway examination was performed using subjective visual assessment and abnormalities, including the presence of stenotic nares, SP elongation, everted tonsils, everted laryngeal saccules, and presence and degree of laryngeal collapse, were noted. Doxapram was not administered during the airway examination. Dogs then underwent upper airway surgery.

Staphylectomy procedures were performed by one ACVS board-certified veterinary surgeon. Folded flap palatoplasty procedures were performed by two other ACVS board-certified veterinary surgeons. With either procedure, the goal of the surgeon was to shorten the palate so that no soft palatal tissue extended beyond the cranial to mid extent of the palatine tonsils. Cut-and-sew staphylectomy was performed using Metzenbaum scissors to sharply resect the redundant palatal tissue, with the nasal and palatal mucosa apposed in a simple continuous pattern with monofilament absorbable suture (4−0 Biosyn™). Folded flap palatoplasty was performed following the technique described by Findji and Dupré [17]. A trapezoidal incision was made in the ventral mucosa of the SP. Rostrally, the incision for the FFP was approximately 1−2 cm caudal to the caudal edge of the palatine bone and dissection extended to the caudal free edge of the SP. Dissection was continued using a monopolar electrosurgery device, and soft tissues were excised to the level of the *palatinus* muscles. The palatinus muscle bellies and the caudal portion of the levator velli palatini muscles were resected, the SP mucosa was then folded on itself, so the nasal SP mucosa was pulled rostrally and closed to the oral mucosa. Mucosal edges were apposed in an interrupted or continuous pattern using monofilament absorbable suture (3−0 or 4−0 Biosyn™ or Monocryl®). The choice of closure pattern and suture was left to the clinician’s preference.

If indicated and determined safe by the clinician on the case, some patients underwent elective surgeries; spay, neuter, and/or urethropexy under the same anesthetic event. Dogs were administered either 0.1 mg/kg dexamethasone sodium phosphate (IV) or 2.2 mg/kg carprofen (SQ) during anesthetic recovery or during the post-operative period to combat iatrogenic inflammation. Dogs recovered in the ICU under respiratory watch.

Post-operatively in the hospital, oxygen supplementation was provided via oxygen cage as needed, as determined by subjective assessment of respiratory effort by the attending clinician or technicians. Post-operative pain was managed in-hospital with methadone (0.1 mg/kg IV) as needed. Dogs received gabapentin and or trazodone post-operatively as needed to facilitate sedation. Metoclopramide (1−2 mg/kg/day continuous rate infusion) was administered to dogs experiencing post-operative regurgitation. Other GI supportive medications, including omeprazole (1−2 mg/kg PO q 12−24 h), pantoprazole (1 mg/kg IV q 12−24 h), maropitant (1 mg/kg IV q 24 h), and cisapride (0.3–0.5 mg/kg PO q 8 h) were administered as needed as determined by the primary clinician. Antibiotics (i.e., cefazolin or ampicillin-sulbactam) were administered as part of a peri-operative antibiotic regime for up to 24 hours, and patients were discharged with carprofen or prednisone as needed for management of post-operative inflammation. Patients were discharged with GI supportive medications (cisapride, omeprazole) as needed. Dogs noted to have dermatitis on physical examination were discharged with a 7–14-day course of cephalexin (22 mg/kg PO q 12 h).

### Follow-up examination

Dogs were scheduled for reevaluation CT examination under sedation 4 weeks post-operatively. Sedation and CT protocols were repeated as described pre-operatively. If tolerated, an airway exam was performed with that sedation protocol. Following the conclusion of the CT scan and airway examination, sedation was reversed with an equivalent dose of atipamezole. Patients were monitored during sedation recovery until they were ambulatory and deemed to be suitable candidates for discharge to their owners.

### Image evaluation and measurements

The CT images were retrieved and reviewed by an American College of Veterinary Radiology (ACVR) resident (CPV) and a board-certified veterinary radiologist (FVG). All images were reviewed in the transverse plane, and multiplanar reformatting was used as needed for interpretation and performing measurements (Fig 1 and 2). Images were primarily reviewed in a soft tissue reconstruction algorithm using a window width of 350 HU and a window level of 50 HU. Bone and lung reconstruction algorithms were also evaluated as needed. Reviewers were allowed to manually adjust window/level settings as necessary. The observers were aware of whether the CT was pre- vs. post-operative, but were blinded to the breed of dog and the type of surgery performed.

**Fig 1:**
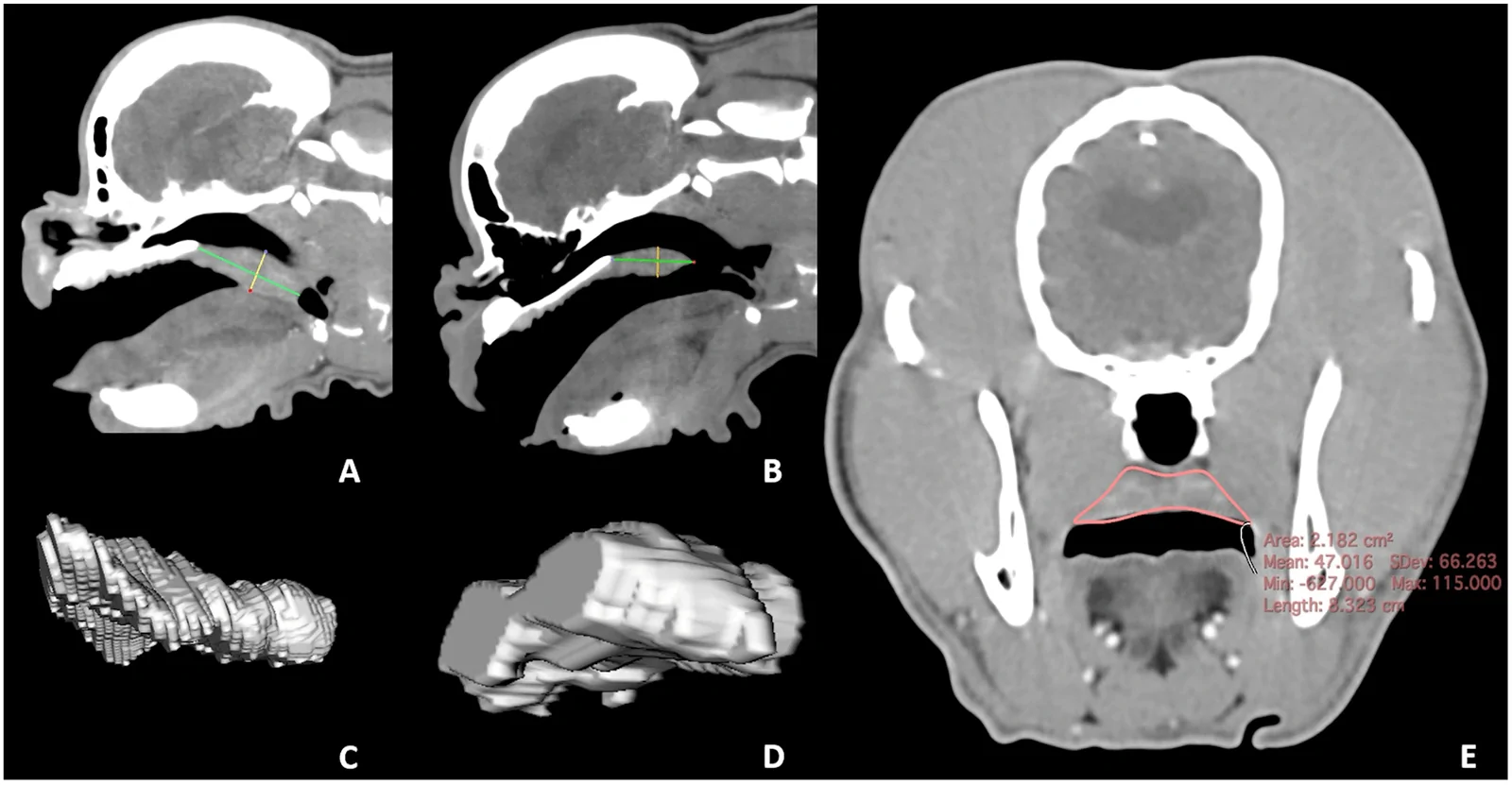
Midsagittal reconstruction view showing soft palate (SP) metric points. Length of the SP (green line); thickness of the SP (yellow line) pre- (A) and post-folded flap palatoplasty (FFP) (B) for the same patient. Sagittal oblique volumetric reconstruction of the SP post-operatively (C), and post-operatively (D) for the same patient. Example of region of interest (ROI) measurement for volumetric calculation (E). Abbreviations: Max, maximum. Min, minimum. SDev, standard deviation.

**Fig 2:**
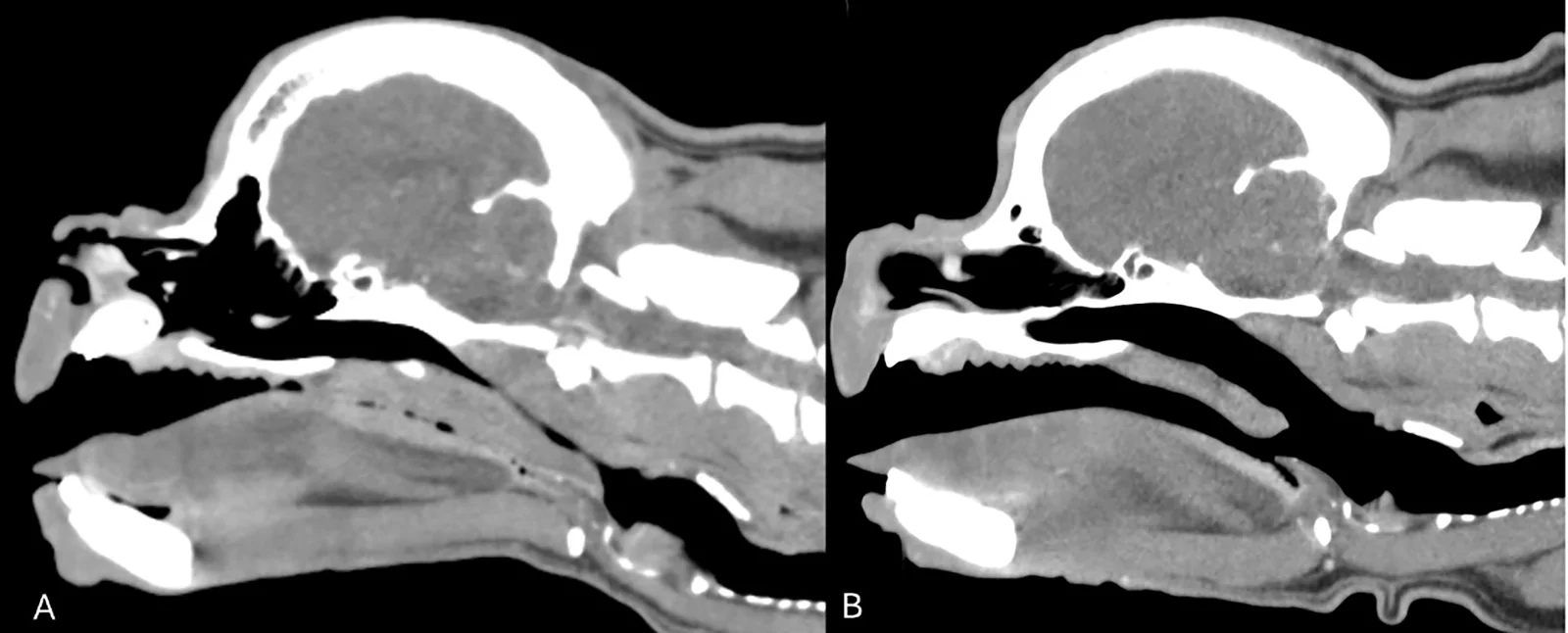
Midsagittal reconstruction view illustrating changes in SP before (A) and after (B) staphylectomy. Abbreviations: SP, soft palate.

Soft palate length was measured on pre- and postoperative CT images according to the technique described by Deprez et al [19], from the caudal end of the hard palate to the caudal end of the soft palate on a sagittal image. Soft palate thickness was determined by identifying the thickest portion of the soft palate and measuring its maximum thickness on a sagittal image. The presence of a thickened SP on CT examination was made based on subjective assessment by the interpreting radiologist when the dorsal surface of the SP was deviating dorsally, causing narrowing of the nasopharynx. No strict measurement criteria were used to objectively determine SP thickening. Horos software was used to estimate the total SP volume through slice-selected areas. The area was calculated on transverse images by drawing regions of interest (ROIs) along the perimeter of the SP. This calculation was performed on every 2 consecutive images that included the selected structure, and segmentation was applied (Fig 1). The ROIs were defined manually, using the technique described by Teodori et al [20]. Soft palate length and thickness measurements, as well as the SP soft tissue volume calculation, were performed by the ACVR resident (CPV) under the supervision of the board-certified veterinary radiologist (FVG). If there were discrepancies between reviewers about the placement of the calipers for measurements or drawing the ROIs, a consensus was reached by discussion and mutual agreement.

### Data collection and analysis

Collected data were grouped into pre-operative, intra-operative, post-operative, and follow-up categories, and recorded in a spreadsheet. Pre-operative data included patient signalment, body condition score, presenting complaints (e.g., snoring, exercise intolerance), physical examination findings, pre-operative CT scan findings, and airway examination findings. Intraoperative data collected included the date of surgery, anesthetic time, procedure time, BOAS surgical procedures performed, other non-BOAS surgical procedures performed, anticholinergic administration, and any anesthetic or operative complications. Post-operative data collected included post-operative medications administered in the hospital or prescribed for discharge, incidence of post-operative regurgitation, incidence of aspiration pneumonia, length of oxygen supplementation (hours), length of hospital stay following surgery (days), and the occurrence of any other post-operative complications. Post-operative complications were grouped into major and minor complications. Minor complications included post-operative vomiting, regurgitation, incisional complications, and aspiration pneumonia. Major complications included the need for tracheostomy or complications resulting in death or euthanasia. Follow-up data collected included date of follow-up examination, time to follow-up (days), need for revision BOAS surgery, post-operative CT imaging findings, follow-up airway examination findings, follow-up clinical signs, and follow-up physical examination findings.

### Statistical analysis

A sample size calculation was performed to determine the number of dogs needed to test our hypothesis with alpha = 0.05 and a minimum power of 0.8. Using a paired t-test size analysis with change in palate thickness as the primary outcome variable, it was determined that a minimum of 7 dogs per group would be needed, given an effect size (anticipated magnitude of difference) of 1.5. Enrollment was planned to conclude once a total of 16 dogs (8 dogs per group) were recruited and completed the study.

Statistical analyses were conducted using JMP®, Version 17 Pro [21]. Pre-operative and post-operative maximum CT measurements of SP length, thickness, and volume are summarized using mean and standard deviation (SD). The changes in pre-operative and post-operative measurements of maximum SP length (cm), maximal SP thickness (cm), and volume (cm^3^) were calculated and summarized using mean and SD. Response data were assessed for normality via visual inspection and found to be normally distributed. A paired t-test was used to test significant differences in post-operative versus pre-operative maximal SP dimensions for both staphylectomy and FFP procedures. Analysis of covariance (ANCOVA) was used to compare post-operative dimensions controlling for pre-operative dimensions between the two procedures. Values of *P* < 0.05 were considered significant.

## Results

### Pre-operative

Eighteen dogs were initially enrolled in this study, 2 of which were later excluded due to failure to return for a post-operative CT scan (Fig 3). Of the 16 included dogs, 8 underwent staphylectomy, and 8 underwent FFP. The study population included French bulldogs (n = 11) and English bulldogs (n = 5). Breeds were relatively evenly distributed among procedure types, with 5 French bulldogs and 3 English bulldogs undergoing staphylectomy, while 6 French bulldogs and 2 English bulldogs underwent FFP. There were 11 males (69%, 6 intact and 5 castrated) and 5 females (31%, all intact) among the study population. Signalment data is further summarized in Table 1. The most common clinical signs experienced among the study population include upper airway noise (n = 14), exercise intolerance (5), and regurgitation (5) (Table 2).

**Table 1:** Characteristics of 16 dogs undergoing either staphylectomy or FFP.

Surgery	Staphylectomy	FFP
**Total Surgeries**	8	8
**Reproductive Status**	FI = 2; MI = 2, MN = 4	FI = 3; MI = 4, MN = 1
**Breed:**		
**French Bulldog**	5	6
**English Bulldog**	3	2
**Mean Age (months)**	21	16.4
**Mean Weight (kg)**	14.7	14.1
**Mean BCS (out of 9)**	6.4	6

Abbreviations: BCS, body condition score. FFP, folded flap palatoplasty. FI, female intact. MI, male intact. MN, male neutered.

**Table 2:** Pre-operative clinical signs among dogs undergoing staphylectomy and FFP.

	Staphylectomy (n = 8)	FFP (n = 8)	Total (n = 16)
**Upper Airway Noise**	8	6	14
**Exercise Intolerance**	2	3	5
**Collapse**	1	0	1
**Vomiting**	2	1	3
**Regurgitation**	3	2	5

Abbreviations: FFP, folded flap palatoplasty.

**Fig 3:**
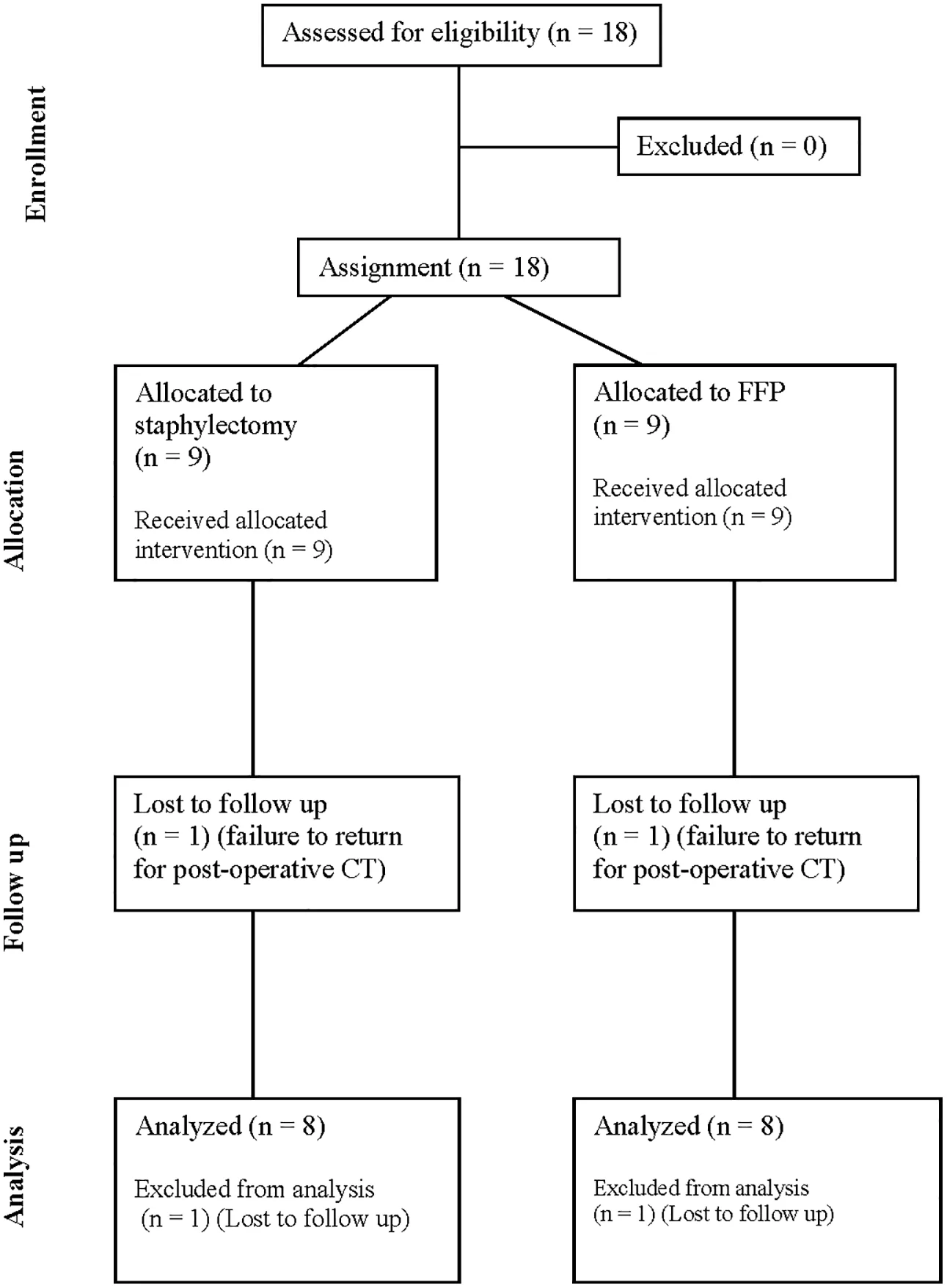
Flow diagram illustrating enrollment, allocation, follow-up, and analysis of patients involved in this study. Abbreviations: FFP, folded flap palatoplasty.

Pre-operative CT findings associated with BOAS included the presence of thickened and elongated SP (n = 16), aberrant nasal turbinates (13), and hiatal hernia (1) (Table 3). Abnormalities were noted in all dogs on pre-operative laryngeal examination, including SP elongation (n = 16), stenotic nares (15), tonsillar eversion (8), laryngeal saccule eversion (8), and laryngeal collapse (8) (Table 4). The distribution of these abnormalities was subjectively similar between groups (Table 4). Of the 8 dogs noted to have laryngeal collapse, 7 were noted to have grade 1 collapse, and 1 was noted to have grade 2 collapse.

**Table 3:** Pre-operative CT findings among dogs undergoing staphylectomy and FFP.

	Staphylectomy (n = 8)	FFP (n = 8)	Total (n = 16)
**Thickened SP**	6	8	14
**Elongated SP**	8	8	16
**Aberrant Turbinates**	7	6	13
**Hiatal Hernia**	1	0	1

Abbreviations: CT, computed tomography. FFP, folded flap palatoplasty. SP, soft palate.

**Table 4:** Pre-operative upper airway exam findings in dogs undergoing Staphylectomy and FFP.

	Staphylectomy (n = 8)	FFP (n = 8)	Total (n = 16)
**Elongated Soft Palate**	8	8	16
**Stenotic Nares**	8	7	15
**Tonsillar Eversion**	4	4	8
**Laryngeal Saccule Eversion**	4	4	8
**Laryngeal Collapse**	4	4	8
**Grade 1**	4	3	7
**Grade 2**	0	1	1

Abbreviations: FFP, folded flap palatoplasty.

### Intra-operative

Eight (50%) dogs underwent staphylectomy, and 8 (50%) underwent FFP. Concurrent airway and non-airway surgeries performed under the same anesthetic episode are summarized in Table 5. No intra-operative surgical complications were noted in any of the cases.

**Table 5:** Concurrent surgical procedures performed in patients undergoing staphylectomy and FFP.

Concurrent Procedure	Staphylectomy (n = 8)	FFP (n = 8)	Total (n = 16)
**Ala-vestibuloplasty**	0	2	2
**Wedge resection rhinoplasty**	8	5	13
**Laryngeal sacculectomy**	1	2	3
**Neuter**	2	5	7
**Urethropexy**	1	0	1

Abbreviations: FFP, folded flap palatoplasty.

### Post-operative

Four out of 16 (25%) dogs received oxygen supplementation post-operatively, with the need for oxygen supplementation determined by subjectively. The average duration of oxygen supplementation was 0.69 hours among dogs receiving oxygen, with a mean duration of supplementation of 1.25 hours in the staphylectomy group (SD = 1.91) and 0.13 hours in the FFP group (SD = 0.35). Three out of 8 dogs undergoing staphylectomy received post-operative oxygen supplementation, while 1 out of 8 dogs undergoing FFP received post-operative oxygen supplementation.

The average duration of post-operative hospitalization, defined as the number of days a patient was hospitalized between anesthetic recovery and discharge, among dogs undergoing either procedure was 0.69 days. The mean duration of hospitalization among the staphylectomy group was 0.38 days (SD = 0.48 days), and the mean duration of hospitalization among the FFP group was 1 day (SD = 0.53 days).

Regarding perioperative and post-operative anti-inflammatory medication administration, 8 dogs received dexamethasone SP IV, and 4 dogs received carprofen SQ. One dog was discharged with a 3-day course of oral prednisone, and 4 dogs were discharged with 3–7-day courses of carprofen. Minor complications, including post-operative regurgitation (n = 2, one in FFP group and one in staphylectomy group) and post-operative bleeding (1) from the rhinoplasty site, were documented in 3 of 16 dogs. No dogs were diagnosed with aspiration pneumonia post-operatively, as evidenced by a lack of reported clinical signs or related findings on 4-week follow-up CT. No major complications such as tracheostomy or death due to cardiopulmonary arrest or euthanasia were experienced among the study population.

### Follow-up

All dogs returned for repeat CT examination of the head, neck, and thorax. The mean duration from surgery to follow-up was 30.4 days (range 20–41 days). At the time of follow-up, owners of 2 dogs that had undergone staphylectomy reported concerns for once-weekly vomiting or regurgitation. The owner of one dog that had undergone FFP reported sneezing and clear nasal discharge. Other than these reported concerns, the remainder of the dogs were reported to be doing well at their re-evaluation.

When comparing pre-and post-operative measurements among dogs undergoing staphylectomy, this procedure resulted in a reduction in SP length (mean 0.91 cm, 95% CI [0.44 cm, 1.39 cm], *P* = .003) and volume (mean 2.81 cm^3^, 95% CI [1.36 cm, 4.27 cm] *P* = .003). Staphylectomy did not result in a SP thinning (mean 0.138 cm, 95% CI [−0.08 cm, 0.356 cm], *P* = .181) (Table 6). Folded flap palatoplasty resulted in reduction in SP length (mean 0.86 cm, 95% CI [0.39 cm, 1.33 cm], *P* = .003), volume (mean 2.54 cm^3^, 95% CI [0.84 cm, 4.24 cm], *P* = .010), and thickness (mean 0.30 cm, 95% CI [0.10 cm, 0.50 cm], *P* = .009) (Table 6).

**Table 6:** Summary of soft palate CT measurements with p-values for ANCOVA comparison between procedures.

	Staphylectomy		FFP	
	Mean	SD	Mean	SD
*Pre-operative soft palate length (cm)*	5.21	0.44	4.51	0.66
*Post-operative soft palate length (cm)*	4.30	0.51	3.65	0.90
*Change in soft palate length (cm)*	−0.91	0.57	−0.86	0.52
*P = 0.9432*				
*Pre-operative soft palate thickness (cm)*	1.33	0.70	1.13	0.41
*Post-operative soft palate thickness (cm)*	1.19	0.56	0.83	0.31
*Change in soft palate thickness (cm)*	−0.14	0.26	−0.30	0.22
***P = 0.0453***				
*Pre-operative soft palate volume (cm^3)*	9.51	4.87	6.18	3.01
*Post-operative soft palate volume (cm^3)*	6.70	3.68	3.64	2.82
*Change in soft palate volume (cm^3)*	−2.81	1.74	−2.54	1.90
*P = 0.4710*				

Abbreviations: ANCOVA, analysis of covariance. CT, computed tomography. FFP, folded flap palatoplasty. SD, standard deviation.

Significant p-values in bold.

There was no difference in the magnitude of reduction of SP length (adjusted mean difference 0.01 cm, 95% CI [−0.33 cm, 0.35 cm], *P* = .943) or volume (adjusted mean difference 0.32 cm^3^, 95% CI [−0.52 cm^3^, 1.16 cm^3^], *P* = .471) between staphylectomy and FFP procedures (Fig 4 and 5, Table 6) when comparing dimensions via ANCOVA. However, FFP did result in greater SP thinning than staphylectomy when compared via ANCOVA, with an adjusted difference in thickness of 0.11 cm (95% CI [0.01 cm, 0.21 cm]) (Fig 6, Table 6).

**Fig 4:**
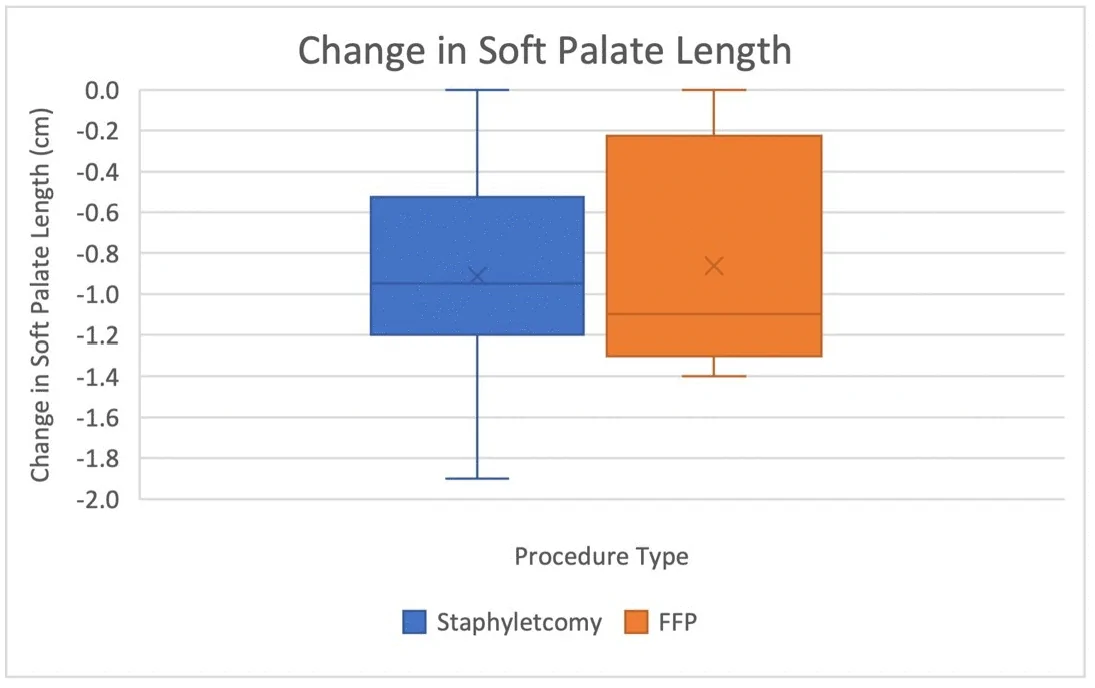
Side-by-side boxplots comparing change in SP length following staphylectomy or FFP. Abbreviations: SP, soft palate. FFP, folded flap palatoplasty.

**Fig 5:**
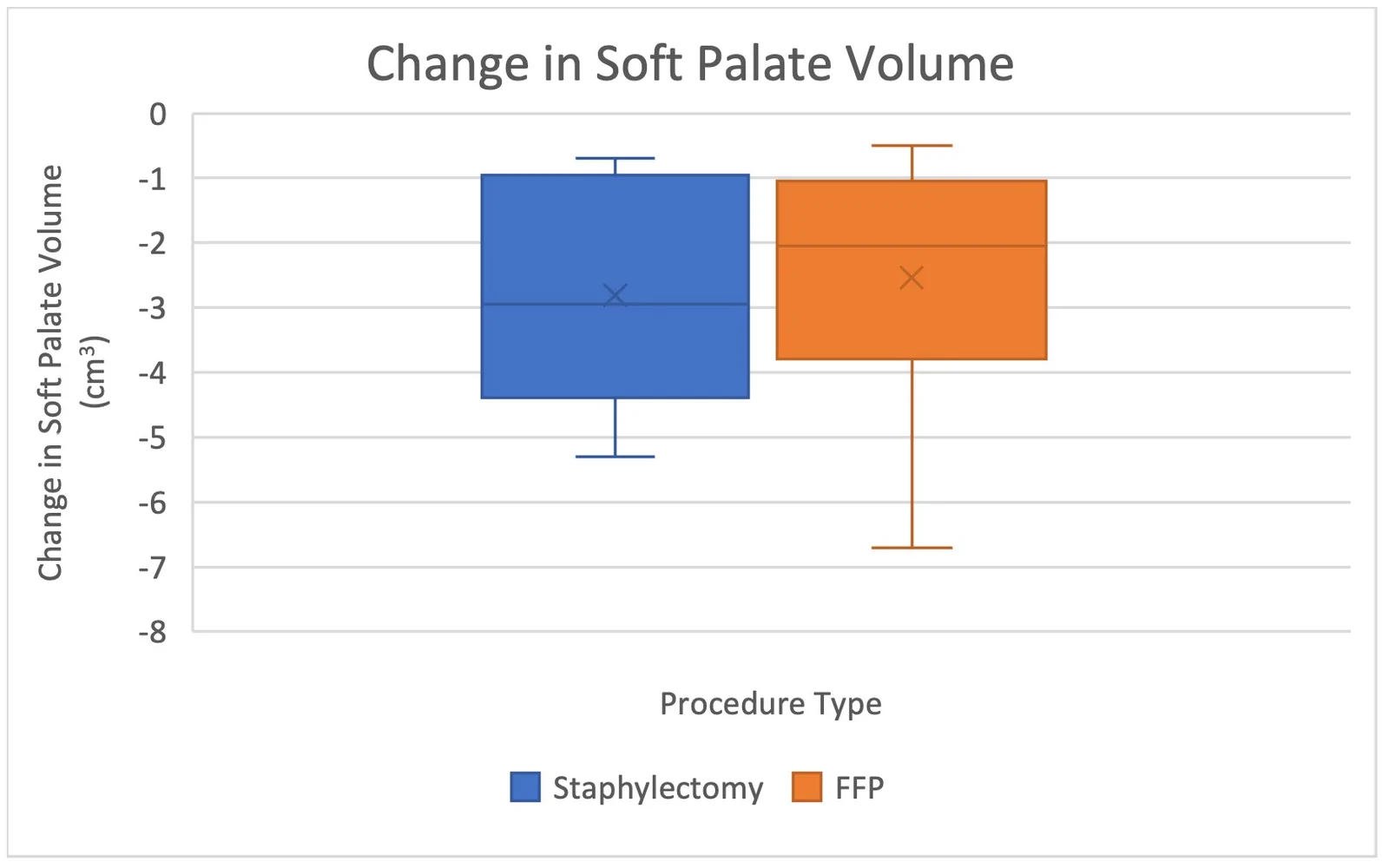
Side-by-side boxplots comparing change in SP volume following staphylectomy or FFP. Abbreviations: SP, soft palate. FFP, folded flap palatoplasty.

**Fig 6:**
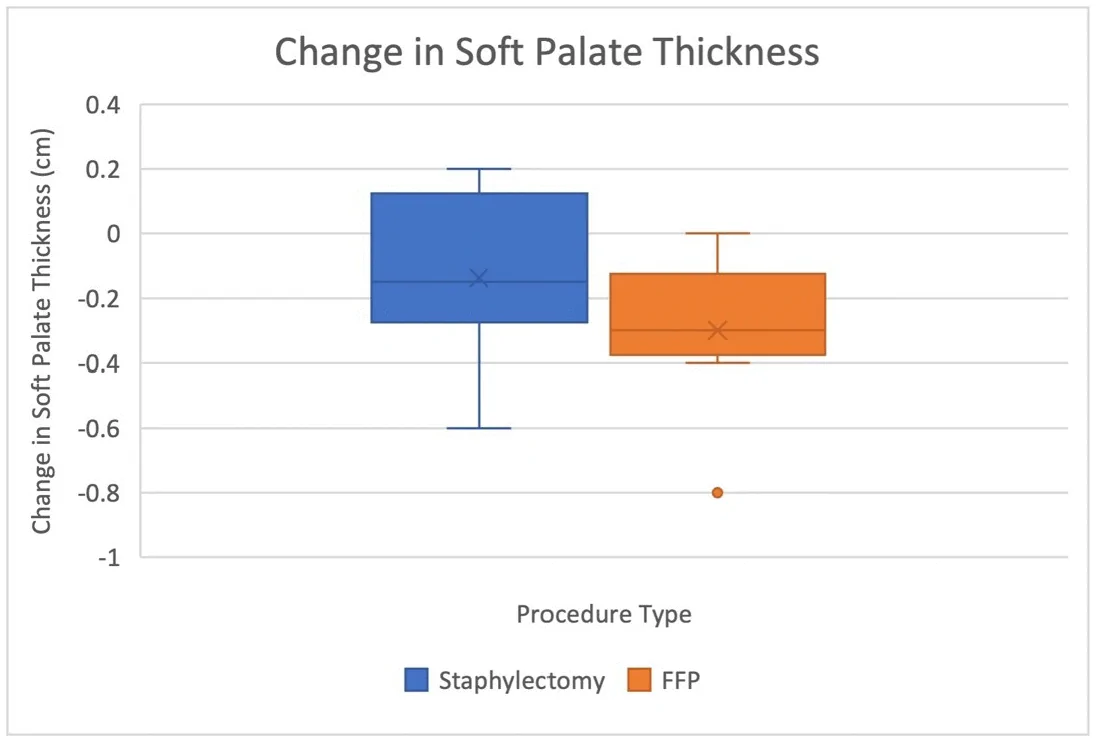
Side-by-side boxplots comparing change in SP thickness following staphylectomy or FFP. Abbreviations: SP, soft palate. FFP, folded flap palatoplasty.

A four-week post-operative airway examination was performed in 8/16 dogs, with 4 examinations repeated in the staphylectomy group, and 4 examinations repeated in the FFP group. A single dog that had undergone staphylectomy underwent revision staphylectomy 231 days after the initial procedure. In the revision procedure, the soft palate was further resected to the level of the caudal third of the palatine tonsils. No dogs undergoing FFP required revision surgery at the time of this study (29−58 months post-surgery), but it was noted in 2 of the cases on follow-up laryngeal examination 4 weeks post-operatively that the FFP site had partially dehisced. The two partially dehisced FFPs had originally been closed by the same surgeon using 3 separate continuous appositional sutures with 3−0 Monocryl. In these cases, the dogs’ owners reported clinical improvement despite the occurrence of dehiscence; thus, revision was not pursued.

## Discussion

This study was successful in its primary objective to compare pre- and post-operative dimensions of the SP in brachycephalic dogs undergoing either staphylectomy or FFP. Sedated CT without endotracheal intubation has been validated as a non-invasive way to obtain objective, reliable measurements of the SP in brachycephalic dogs [18,19]. The results of this study indicated that both staphylectomy and FFP surgeries were effective at reducing SP length and volume when comparing pre- and post-operative CT measurements. However, FFP reduced mean SP thickness by approximately 26%, while staphylectomy did not demonstrate any thinning.

The finding that both staphylectomy and FFP were successful in reducing SP length and volume, with no significant difference in efficacy between procedures, supports the effectiveness of FFP in SP length reduction. This is accomplished via rostral retraction of the caudal edge of the SP, as proposed by Findji and Dupré, who found that 97.5% of 55 dogs that underwent FFP experienced an improvement in post-operative clinical respiratory function [17]. The lack of difference in post-operative length reduction between staphylectomy and FFP indicates first that FFP is as successful in shortening the elongated SP, and second that FFP does not objectively result in excessive SP shortening as compared to staphylectomy, as has been a concern of some authors for increasing nasopharyngeal reflux events [1,17,22,23].

Folded flap palatoplasty resulted in SP thinning, consistent with the goal of the FFP procedure being to reduce SP thickness to relieve both nasopharyngeal and oropharyngeal obstruction [17]. One prior study did report SP thinning 3 months following staphylectomy [24], which had not been observed in two other studies examining SP dimensions post staphylectomy [25,26]. However, in the aforementioned study, pre- and post- operative SP dimensions were not compared to determine the statistical significance of the degree of thinning, and thus the relevance of the identified thinning may remain questionable. The reason for this observed thinning was proposed to be due to resolution of edema following relief of airway obstruction. In our study, staphylectomy did not result in statistically significant thinning of the SP. Therefore, FFP may be a more appropriate choice for surgical management in dogs found to have a thickened SP in addition to SP elongation. This is particularly relevant as thicker SPs have been significantly associated with the severity of BOAS, and thus, FFP should be considered when managing dogs more significantly affected by BOAS [8]. Soft palate thickness may be difficult to assess via airway examination alone; therefore, CT examination of SP dimensions and/or nasopharyngoscopy may be considered as an element of pre-operative planning prior to upper airway surgery to determine whether FFP or staphylectomy is indicated.

In contrast to prior studies by Grimes et al., Timmermans et al., and Farrell et al. that identified no significant difference in soft palate dimensions, [24–26], this study did indicate superior SP thinning with FFP compared to staphylectomy. One recent study by Grimes et al. noted no significant difference in the SP thinning between staphylectomy and FFP [25], which differed from the findings of this present study. Potential stated limitations of the study by Grimes et al. that may explain the difference in results include an acknowledgement that surgeons performing FFP had limited experience with the procedure. Given a potential learning curve, the success of the procedure in thinning the SP may have been limited. Additionally, authors of this study acknowledged that dogs severely affected by BOAS or with excessively thick SPs were not included, potentially limiting the degree of SP thinning that could be achieved. Similarly, a study by Timmermans et al. included pugs in the study population [26], which were likely to have thinner SPs pre-operatively, thereby limiting the effect of FFP. All three studies had small sample sizes that may have resulted in type II error and failure to recognize small differences in SP dimensions.

While our study illustrated that FFP is theoretically advantageous when compared to staphylectomy, considering the ability to simultaneously shorten and thin the SP, possible drawbacks of the procedure include that some surgeons may find FFP to be a more technically demanding and time-consuming procedure, as well as the potential unfolding of the palate if the incision is to dehisce. Flap ischemia or excessive tension may contribute to surgical site dehiscence in these cases and lateral releasing incisions while performing the FFP can help with tension, though this technique was not performed in our study. A study by Khoo et al. found a 36% incidence of incisional dehiscence in dogs that underwent FFP [27]. The potential benefit of FFP may be compromised by the risk of unfolding and subsequent recurrence of soft palate elongation and worsening of clinical signs in the case of dehiscence [27]. In contrast, dehiscence of a staphylectomy site will likely heal rapidly by second intention. Two out of 8 dogs undergoing FFP were found to have experienced partial incisional dehiscence. The two partially dehisced FFPs had originally been closed using 3 simple continuous suture lines with 3−0 Monocryl. Surgeons may also consider closing FFPs using an interrupted pattern to potentially reduce dehiscence risk. Though partial incisional dehiscence occurred in these two cases, CT measurements did not indicate a lack of length reduction post-operatively associated with FFP in these dogs, indicating that the extent of dehiscence was not so severe that the SP remained elongated among the FFP group. Regardless of partial dehiscence, these dogs experienced improved clinical signs without any concerns reported by their owners, and neither of these dogs required revision surgery. These dogs remained included in measurement analysis in this study given the lack of standardized airway examination among the study population at recheck exam, and to reflect a potentially realistic outcome which may be expected in clinical practice.

Complications in this study were rare and included only minor complications in 3/16 cases. However, as airway examinations were not consistently performed and documented post-operatively, these numbers were not included in the statistical analysis. The low incidence of complications in this study supports the conclusion of Findji and Dupré, that FFP is a safe and effective procedure [17].

One dog that underwent staphylectomy required a revision staphylectomy 231 days following the procedure. The dog requiring revision was 6 months of age at the time of the original procedure and severely affected by BOAS. The need for revision may have been due to a more cautious resection given the young age or potentially because of continued palatal growth. The optimal age for palate surgery remains unclear.

There are several limitations of this study, including a limited sample size and a lack of standardization of post-operative management. The small sample size predisposes this study to potential type II error, particularly when it comes to detecting small differences in measurement changes between the two procedures. Given the high rate of clinical improvement reported for both procedures [17,28,29], the detection of differences in clinical outcome, or small differences in palate dimension between procedures, may require a significantly larger sample size and remains an avenue for further investigation.

Administration of anti-inflammatory medications (dexamethasone SP, prednisone, and carprofen) was not standardized in this study, and could contribute to changes in soft palate dimensions. However, any anti-inflammatory medications that were prescribed were discontinued within 7 days of discharge. Therefore, the impact of these medications on palate dimensions at the 4-week postoperative time point is questionable.

Finally, 4-week post-operative airway examinations were not documented in all dogs. Therefore, follow-up airway examination results were not fully presented in this study. Consistent, well-documented airway exams would allow further assessment of potential post-operative incisional complications, which is an important factor when considering outcomes and success of staphylectomy and FFP.

A further possible limitation of this study is the method of measurement for SP thickness. In this study, mid-sagittal SP thickness was measured at the point of maximal dimension, as this was thought to be most clinically relevant. This differs from the methodology of prior studies comparing pre- and post-operative SP dimensions at various points along its length [24–26], and may contribute to a difference in results between studies.

An additional potential limitation to this study would be the exclusion of dogs with grade III laryngeal collapse. Though this decision was made for patient safety reasons, it may have eliminated a population of patients more significantly affected by BOAS. These dogs may have more significant soft palate hyperplasia and edema as a consequence of the severity of their obstruction and may therefore be more likely to benefit from FFP. The exclusion of these cases with potentially larger pre-operative palate dimensions may have tempered the degree of reduction in SP dimensions observed. Therefore, it is important to consider that dogs with high grade laryngeal collapse may be more likely to benefit from FFP as compared to staphylectomy, though this was not specifically examined in this study.

In this study, each dog included was found to have a subjectively thick SP. However, it is worth noting that this procedure may not necessarily be indicated in dogs that have elongated but thin SPs. There is no strict objective measure or consensus of what defines a SP as thickened on CT examination, and in this study, this finding was reported based on subjective assessment by the radiologist. One prior study did define mild, moderate, and marked thickening of the SP based on the percentage of nasopharyngeal narrowing, though this definition has not been otherwise validated [30]. A simple, objective, and repeatable CT measurement definition or grading of SP thickness may lend to greater clarity in the diagnosis of a thickened SP.

In conclusion, staphylectomy did not result in SP thinning, while FFP did result in SP thinning. Therefore, our hypothesis was accepted. Evaluating SP thickness prior to surgery via CT scan and considering thickness, in addition to length, when choosing which surgical procedure to perform for treatment of an elongated SP may be beneficial when deciding which surgical procedure to perform.
